# Work Participation among Women and Men in Sweden: A Register Study of 8.5 Million Individuals

**DOI:** 10.3390/ijerph18094642

**Published:** 2021-04-27

**Authors:** Katriina Heikkilä, Ellenor Mittendorfer-Rutz, Kristina Alexanderson, Marianna Virtanen

**Affiliations:** 1 Division of Insurance Medicine, Department of Clinical Neuroscience, Karolinska Institutet, SE-171 77 Stockholm, Sweden; ellenor.mittendorfer-rutz@ki.se (E.M.-R.); kristina.alexandersson@ki.se (K.A.); 2 School of Educational Sciences and Psychology, University of Eastern Finland, FI-80101 Joensuu, Finland; marianna.virtanen@uef.fi

**Keywords:** working life, sustainable work, sex differences, register data

## Abstract

Observational research studies from various countries suggest that women’s working patterns across the life course are often fragmented compared to men’s. The aim of our investigation was to use nationwide register data from Sweden to examine the extent to which generation and time of entry to the work force explain the sex differences in work participation across the life course. Our analyses were based on individual-level data on 4,182,581 women and 4,279,571 men, who were 19–69 years old and resident in Sweden in 1995, 2000, 2005, 2010, or 2015. Data on income and number of net days on disability pension, obtained from multiple linked registers, were used to ascertain each individual’s main activity (in paid work, on disability pension, and not in paid work) each year. Years in paid work and on disability pension were calculated as the sums of years spent in either of these states from age 19 to 69 years. We used negative binomial regression to model the associations of generation and baseline year with years in paid work and years on disability pension. All models were run separately for women and men, with the duration of follow-up constrained to one, to account for the different follow-up times between individuals. Overall, the number of years in paid work across the life course was larger among men than women, and men entered into the workforce earlier. The difference between women and men was similar across generations and time periods. Adjustment for education, income, number of children aged <18 years living at home, country of birth, and the type of residential area had minimal impact on the estimates. Our findings suggest that women spend fewer years in paid work across the life course than men, highlighting the need for continued efforts to close the gender gap in work participation.

## 1. Introduction

Labour market participation is important to individuals, families, and the wider society. Paid work enables workers to support themselves and their families and be financially independent [1,2]; afford housing, healthcare or education for the worker or their dependents and, in so doing, positively impact their health and quality of life [3] Participating in paid work outside of the home also can offer opportunities to increase aspects of social capital, such as civic and social participation [4]. Work defines a worker’s role in society, in many instances, and offers them a means to interact with others and engage in and influence society in many different arenas, ranging from trade unions to professional organisations to informal, workplace-based networks.

Participating in paid work has varied considerably over time and across geographical areas, and it is influenced by a number of interconnected factors at individual-, workplace- and societal-levels. One important factor influencing work participation is sex [5]. Worldwide, women’s and men’s work patterns differ; for example, women’s working lives are typically shorter and more fragmented than men’s, with unemployment, temporary work contracts, and shift work being more common among women than men in practically all countries [5,6]. 

Theories of mechanisms underlying sex differences in work participation include sex discrimination and labour market segregation, which can steer women and men toward types of work that differ in terms of socioeconomic gains or health implications, or preclude women from engaging in paid work to the same extent as men [7,8]., A systematic review of mainly European observational studies shows that women, particularly those from socioeconomically disadvantaged circumstances, tend to exit the labour market by retirement earlier than men [9], for instance. Register-based research from the Nordic countries has shown that women, on average, have higher rates of sickness absence and disability pension than men [10,11,12,13,14], whereas a study conducted in Spain found disability retirement being more common among men [15]. There also is research to suggest that poor self-rated health and a high prevalence of musculoskeletal and psychiatric diseases among women may lead them to exit the labour market earlier than men [12,16,17].

Other theories include gender division of labour in the household and family–work conflict, which can put women at a disadvantage in the labour market if they undertake the majority of unpaid household and care work in the family, with males performing the main income earning work [8,18,19]. These theories are supported by findings from longitudinal studies of working lives which suggest that, on average, women undertake a large proportion of childcare and domestic responsibilities [20,21], which is reflected by the relatively high frequency of part-time work among partnered women in many countries [21] and women spending more time on family and care leave than men in countries with extensive provisions for these types of leave [21,22].

The aim of our investigation was to examine work participation across the life course of the working life among men and women, and to investigate whether work participation differed by generation or time period. To ensure the generalisability of the findings, we used individual-level data from multiple linked national registers of all Swedish residents aged 19–69, extracted between 1995 and 2015.

## 2. Materials and Methods

### 2.1. Data Sources

We used anonymised individual-level data from the Longitudinal Integration Database for Health Insurance and Labour Market Studies (LISA) [23] to identify the study population and obtain information on sociodemographic characteristics. Numbers of days in receipt of sickness absence benefits and on disability pension were ascertained from Micro-Data for Analysis of the Social Insurance System (MiDAS) [24] and deaths from the Swedish national Cause of Death Register. Individual-level data from these registers were linked by Statistics Sweden, using the unique personal identity numbers assigned to all residents in Sweden [24]. The research was approved by the Regional Ethical Review Board, Stockholm, Sweden

### 2.2. Study Population

The study population comprised men and women who were 19–69 years old, resident in Sweden on 31st December the year preceding one of the baseline years (1995, 2000, 2005, 2010, or 2015), and who were alive and registered as living in Sweden on 31st December of the baseline year and one year following the baseline year. Thus, each individual contributed a minimum of one year of data to the study. Individuals up to the age of 69 were included in our analyses because, in Sweden, old-age pension can be taken from the age of 61 to 67 years (or even later at the employers’ discretion).

### 2.3. Exposures

The main exposures in our analyses were sex (women or men), generation (Traditionalists, born before 1946; Baby boomers, born 1946–1964; Generation X, born 1965–1976; and Generation Y, born 1977–1995), and time period (baseline year: 1995, 2000, 2005, 2010, or 2015).

### 2.4. Outcomes

The main outcomes were the overall number of years spent in paid work and on disability pension, from the baseline year to the end of follow-up. These were calculated using data on income from work (from LISA) and number of net days on disability pension (from MiDAS). One of three states (in paid work, on disability pension, or not in paid work) was ascertained for each individual for each year, based on their principal activity during that year. Individuals were defined as being in paid work if they received at least half of their annual income from work and were not registered to receive disability pension for more than ≥183 net days during the year in question. Individuals were defined as being on disability pension if they received a disability pension for ≥183 net days per year, regardless of whether they had other income from work or benefits. Not being in paid work was defined as receiving less than half of their annual income from work and not receiving any disability pension. This category included individuals in diverse circumstances, e.g., full-time students and old age pensioners, and it was not the focus of the current investigation.

### 2.5. Covariates

Covariates were factors known to be associated with sex, age, and working life outcomes [11,25,26,27,28,29]. All covariates were ascertained from LISA on the baseline year and analysed as categorical variables. The covariates included in the minimum-adjusted models were achieved education (compulsory: <10 years, tertiary, e.g., high school or equivalent: 10–12 years, higher, e.g., university or equivalent: >12 years) and income quintile (disposable income in Swedish kronor/per year, calculated across the whole study population). Multivariable-adjusted models were additionally adjusted for the number of children aged <18 years living in the household (none, 1, 2, 3 +), country of birth (Sweden, other Nordic country, other EU25, or country outside the EU), and type of residential area in relation to population density (city, town, or rural area).

### 2.6. Statistical Analyses

All individuals contributed time to the analyses until the year when they turned 69, died, emigrated from Sweden, or the study follow-up ended (31 December 2016), whichever occurred first. Years spent in paid work, on disability pension, and not in paid work were calculated for each individual. These were summarised as medians and interquartile ranges (IQRs) for women, men, generations, and baseline years. We used negative binomial regression to estimate incidence rate ratios (IRRs) and their 95% confidence intervals (CIs) to quantify the associations of generation and baseline year with the count outcomes (years in paid work and years on disability pension). This modelling approach was chosen because the counts were over-dispersed and contained large proportions of zero-values. All models were run separately for women and men, with the duration of follow-up constrained to one, to account for the different follow-up times between individuals. All analyses were conducted using Stata SE 16 (Stata Corporation, College Station, Texas, US).

## 3. Results

Ultimately, 4,182,581 women and 4,279,571 men, aged 19–69 years, were included in our analyses (Table 1). Overall, the level of achieved education was slightly higher among women, whereas the income from work was higher among men. A slightly larger number of women than men, on average, had one or more children aged <18 years living at home. The majority of the study population (82.5%) were born in Sweden.

**Figure 1:**
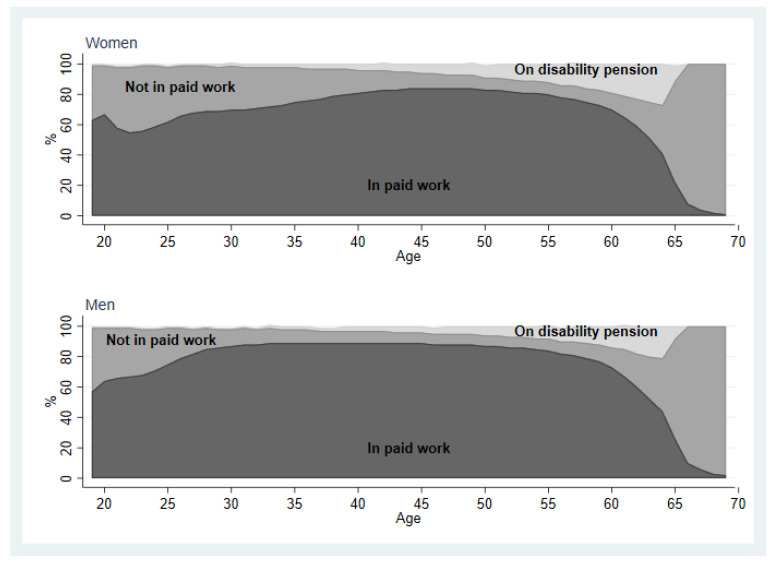
Work participation across the life course, among women and men aged 19–69 in Sweden from 1995–2015.

Overall, men spent a greater number of years in paid work than women between age 19–69 and, conversely, the number of years spent not in paid work or on disability pension was greater among women than men (Figure 1). Generally, women’s entry into the labour market occurred later than men’s: approximately 80% of men and just over 60% of women were in paid work in their late twenties. Around the age of 40–45 years, approximately 80% of both women and men were in paid work. The median number of years worked was 1–4 years less among women than men across generations and baseline years (Table 2).

The associations of generation and baseline year with working life outcomes are shown in Table 3. Compared to Baby Boomers (born 1946–1964), the number of years in paid work were greater among the women as well as the men of Generation X and less among subsequent generations, even when the shorter duration of follow-up for the later generations was considered. Compared to those in the workforce in 1995, women and men who entered the workforce in 2000 worked fewer years and those who entered in 2005, 2010, or 2015 spent a greater number of years in paid work. Adjustment for the level of education, income, number of children <18 years of age living in the household, country of birth, and type of residential area did not markedly change these estimates. To examine the extent to which family composition is associated with the differences in labour market participation between women and men, we undertook sensitivity analyses limited to data from individuals with no children aged 18 years or younger living in the household. The findings from these analyses, provided in the Online Supplement, Supplementary Table S3, suggest that although the sex differences in work participation were fewer among those with no children living in the household, the gender gap was still evident in this group.

Table 4 shows that the number of years in paid work was generally less among women than men, but this association varied by generation and, among the oldest and the youngest generations, women in fact spent a larger number of years in paid work.

Associations of generation and baseline years with the number of years on disability pension are shown in the Online Appendix, Supplementary Table S1. Compared to Baby Boomers, earlier and later generations spent fewer years on disability pension. Similar to years in paid work, both women and men who entered the workforce in 2000 spent fewer years on disability pension compared to those in the workforce in 1995. However, those who entered the workforce in 2005, 2010, or 2015 accumulated more years on disability pension, independently of covariates. Supplementary Table S2 suggests that women spent fewer years on disability pension, overall and across generations.

## 4. Discussion

The gender gap in work participation is a global phenomenon. Despite narrowing of the gender gap across the past decades, women’s labour force participation still lags behind men’s practically everywhere. Overall, in Europe, women’s work participation increased from cs. 55% in the mid-1990s to circa. 66% in 2008 [30]. Regarding the Nordic countries, the gender gap in work participation is considerably smaller than elsewhere in Europe, but it has remained relatively unchanged from the mid-1990s to 2008 [30,31]. Our results indicate that across the life course, men in Sweden still spend more years in paid work than women, although this pattern varied somewhat by generation. Adjustment for sociodemographic factors (level of education, income, number of children aged <18 years living at home, country of birth, and the type of residential area) had minimal impact on the association estimates, suggesting that these factors do not explain a marked proportion of the variation in work participation between women and men. Our observations highlight the need to continue efforts to reduce the gender gap in work participation.

The mechanisms underlying labour market participation among women and men are likely to be manifold and interlinked: they can be a result of explicit employment, taxation and welfare policy design, or represent an unintended consequence of fragmented policy design in multiple areas [32]. Personal choice also can affect individual employment decisions. During our study, women and men of Generation X (born in 1965–1976) spent more years in paid work than Baby Boomers (those born in 1946–1964), an observation that may relate to the latter generation retiring earlier, prior to the concerted policy efforts to extend working lives into older ages. Taking a similar vein, our observations that the women of the oldest generation (born before 1946) and the youngest generations (born after 1996) worked slightly more years than men may reflect women typically living longer than men and a larger proportion of men than women undertaking military service (7–12 months in Sweden), respectively.

Findings from many studies, including ours, support the theory that one key driver of the fragmentation of women’s working lives is the large proportion of childcare and domestic responsibilities undertaken by women [20,21,33]. Indeed, previous research has shown that although male use of parental leave is higher in Sweden than in many other countries, the majority of the leave is still taken by women [31,33,34]. Regarding our study population, women were less likely to be mainly in paid work in their twenties and thirties than men: approximately 80% of men were working in their late twenties, whereas women reached this proportion around the age of 40–45 years. The observation that a lower proportion of women than men in their early twenties had their main income from work may reflect women spending longer periods in full-time education (with higher education student benefits as their main source of income) or on family-related leave (with maternity or childcare benefits as their main source of income). However, our sensitivity analyses, limited to data from individuals with no children aged 18 years or younger living in the household, suggest that although the sex differences in work participation were smaller in this group than in the overall study population, the gender gap still was evident. Taken together, these findings suggest that gender division of household labour is likely to be one, but not the sole, mechanism explaining the sex differences in work participation in our study.

It is possible that the sex differences in work participation are related to fixed term contracts and/or part-time work being more common among women than men. More women than men work part-time in central and northern Europe, on average, including Sweden [30]. During our investigation, individuals were defined as being in paid work if they received at least half of their annual income from work (as opposed to disability pension or benefits/allowances, e.g., sickness absence benefit, unemployment benefit, maternity allowance, or student allowance). Part-time workers were defined as being in paid work or not being in paid work, depending on what proportion of their income they received from work. Thus, the overall work participation across the life course was less for individuals who worked part-time or on short term contracts and received most of their annual income from sources other than work.

Another important determinant of work participation is the type of work: for example, both female and male white-collar workers are more likely than blue-collar workers to continue working into old age [35]. Our analyses were adjusted for achieved education, income, country of birth, or type of residential area, but we had no data available on specific occupations and, thus, were unable to further investigate the degree to which work participation was impacted by the type of work women and men were engaged in, or the role of gender segregation of labour in our study population.

Patterns of being in paid work toward the end of the working life were, to a great extent, similar in women and men, although women spent slightly less time on disability pension compared to men. Generally, individuals moved from work or disability pension to old-age pension around the age of 63–66 years. These patterns are in contrast to some previous findings, which suggest that women were more likely than men to be on disability pension during the final years of their working lives, thus exiting the labour force earlier than men [36]. Our observations may reflect an effect of the disability pension reform, which also has been observed in other studies [35]: the change in the legislation in Sweden in 2008 levelled the sex differences in the disability pension when fixed term disability pensions were terminated and people who, prior to the reform, were on fixed term disability pensions now tend to spend the final years of their working lives on long-term sick leave.

The evidence for specific health issues as determinants for sex differences in work participation is unclear. Some study findings point to poor self-rated health and a high prevalence of musculoskeletal diseases and mental health disorders among women, leading them to exit the work force earlier than men [12,16,17], yet several other investigations have found no clear evidence for specific family, health, or work-related factors accounting for differences in the duration or patterns of working lives between women and men [9,11,13]. We did not examine the potential impact of specific diseases on the differences in work participation between women and men. It is possible that health consequences of childbirth limit some women’s labour market participation, particularly during the early working life, but it is unlikely that these would impact the work participation among women at population-level. Taking a similar vein, previous research has shown that severe menopausal symptoms can have an adverse influence on some women’s ability to work, but the population-level significance of this is unclear [37].

Spending fewer years than men in paid work across the life course may have implications to women’s health if women who do not work or work part-time have reduced or fragmented access to occupational healthcare. This may be particularly relevant to women with diseases or conditions that would be best managed in occupational healthcare or in regular contact with the same healthcare provider.

### Strengths and Limitations

A major strength of our investigation is that we used a large, nationally representative set of individual-level data on all working-age women and men in Sweden. Since our data were obtained from nationwide registers, with near-complete coverage and good quality data, they have not been influenced by response biases or attrition. Due to the size of our analytical dataset, we were able to undertake subgroup analyses and produce precise estimates which are generalisable to the working age population in Sweden. Since working conditions and social security systems differ between countries, however, our findings may not be directly generalisable to other countries.

It is a limitation that working life outcomes (years in paid work and years on disability pension) were based on each individual’s principal activity in each year. Consequently, the real working life patterns, for some individuals, may be more complex than we estimated. However, it is unlikely that such misclassification in the patterns of working life differs by sex (the key comparison in our analyses) and, thus, it is more likely to have reduced the precision of our estimates, rather than led to significant bias. Adjustment for level of education, income, number of children aged <18 years living at home, country of birth, and the type of residential area had minimal impact on the association estimates, suggesting that these factors do not explain a marked proportion of the variation in work participation between women and men. However, it is possible that these covariates did not fully capture the sociodemographic and work characteristics that explain the difference in work participation between women and men. Thus, we cannot preclude the possibility that our observations have been influenced by residual confounding from unmeasured or unknown work-related factors.

## 5. Conclusions

Our findings suggest that women in Sweden spend fewer years in paid work across the life course than men. These observations highlight the need to continue the efforts to close the gender gap in work participation.

## Figures and Tables

**Table 1:** Sociodemographic characteristics of the study population (*n* = 8,462,152).

Baseline Characteristics (*n*, %)	Women (*n* = 4,182,581)	Men (*n* = 4,279,571)	*p*
**Generation**			
Traditionalists (pre-1946)	935,073 (50.5)	914,964 (49.5)	
Baby Boomers (1946–1964)	1,179,715 (49.1)	1,221,028 (50.9)	
Generation X (1965–1976)	502,505 (49.2)	827,210 (50.7)	
Generation Y (1977–1995)	1,168,332 (49.1)	1,213,639 (51.0)	
Generation Z (1996-)	96,956 (48.6)	102,730 (51.5)	<0.0001
**Baseline year**			
1995	3,596,348 (49.3)	3,694,314 (50.7)	
2000	277,055 (49.6)	281,868 (50.4)	
2005	80,601 (52.0)	74,386 (48.0)	
2010	110,624 (49.7)	111,986 (50.3)	
2015	117,953 (50.2)	117,017 (49.8)	<0.0001
**Level of achieved education**			
Compulsory: <10 years	1,071,624 (47.0)	1,210,839 (53.1)	
Tertiary: 10–12 years	2,263,177 (49.9)	2,271,472 (50.1)	
Higher: >12 years	847,780 (51.5)	797,260 (48.5)	<0.0001
**Income quintile (disposable income, SEK/year)**			
1st (lowest)	1,764,024 (52.5)	1,596,679 (47.5)	
2nd	707,078 (59.3)	485,416 (40.7)	
3rd	732,442 (56.6)	562,535 (43.4)	
4th	577,906 (44.1)	734,114 (56.0)	
5th (highest)	401,131 (30.8)	900,827 (69.2)	<0.0001
**Number of children aged <18 years living at home**			
None	2,457,758 (47.7)	2,695,771 (52.3)	
1	850,753 (52.3)	776,303 (47.7)	
2	606,364 (52.1)	557,754 (47.99)	
3+	267,706 (51.7)	249,743 (48.3)	<0.0001
**Country of birth**			
Sweden	3,433,012 (49.2)	3,550,708 (50.8)	
Other Nordic country	160,167 (55.0)	131,336 (45.1)	
Other EU 25	126,861 (49.6)	128,796 (50.4)	
Outside the EU	462,541 (49.7)	468,731 (50.3)	<0.0001
**Type of residential area**			
City	1,517,919 (50.0)	1,521,024 (50.1)	
Town	1,487,415 (49.6)	1,514,315 (50.5)	
Rural area	1,177,247 (48.6)	1,244,232 (51.4)	<0.0001

**Table 2:** Person-years in paid work and on disability pension, with length of follow-up, among women and men aged 19–69 in Sweden from 1995–2015.

	Years in Paid Work ^1^			Years on Disability Pension ^2^			Years of Follow-Up	
	Median (IQR)			Median (IQR)			Median (IQR)	
	Women	Men	*p*	Women	Men	*p*	Women	Men
Generation (birth year)								
Traditionalists (pre-1946)	2 ( < 0.5–8)	3 (0–9)	<0.0001	4 (2–7)	4 (2–7)	<0.0001	14 (9–18)	15 (10–18)
Baby Boomers (1946–1964)	18 (12–21)	19 (13–22)	<0.0001	9 (4–15)	9 (4–15)	<0.0001	22 (22–22)	22 (22–22)
Generation X (1965–1976)	16 (11–19)	20 (14–22)	<0.0001	6 (3–12)	8 (3–15)	<0.0001	22 (22–22)	22 (22–22)
Generation Y (1977–1995)	6 (3–10)	7 (4–12)	<0.0001	4 (2–7)	4 (2–7)	<0.0001	22 (17–22)	22 (17–22)
Generation Z (1996-)	1 (1–2)	1 ( < 0.5–2)	<0.0001	1 (1–1)	1 (1–2)	0.0147	17 (17–17)	17 (17–17)
**Baseline year**								
1995	11 (4–18)	13 (5–20)	<0.0001	6 (2–11)	2 (2–10)	<0.0001	22 (20–22)	22 (20–22)
2000	2 (1–5)	2 (1–4)	0.0482	3 (1–8)	3 (1–7)	<0.0001	17 (17–17)	17 (17–17)
2005	7 (3–10)	8 (4–11)	<0.0001	4 (2–9)	5 (2–9)	0.08	12 (12–12)	12 (12–12)
2010	4 (2–6)	6 (3–7)	<0.0001	3 (2–5)	3 (1–5)	0.3	7 (7–7)	7 (7–7)
2015	2 ( < 0.5–2)	2 (1–2)	<0.0001	2 (1–3)	2 (1–3)	0.9	2 (2–2)	2 (2–2)
All	10 (3–18)			5 (2–10)			22 (17–22)	

^1^ At least half of annual income from paid work and not on disability pension for ≥183 net days per year. ^2^ On disability pension for ≥183 net days per year, regardless of sources or amount of income.

**Table 3:** Associations of generation and baseline year with years in paid work, among women and men aged 19–69 in Sweden in 1995–2015.

	IRR (95% CI) ^1^					
	Mutually-Adjusted ^2^		Minimum-Adjusted ^3^		Multivariable-Adjusted ^4^	
	Women	Men	Women	Men	Women	Men
Generation (birth year)						
Traditionalists (pre-1946)	0.42 (0.42–0.42)	0.42 (0.42–0.42)	0.45 (0.45–0.45)	0.42 (0.42–0.42)	0.47 (0.47–0.47)	0.44 (0.44–0.44)
Baby Boomers (1946–1964)	1 (ref. cat.)	1 (ref. cat.)	1 (ref. cat.)	1 (ref. cat.)	1 (ref. cat.)	1 (ref. cat.)
Generation X (1965–1976)	0.98 (0.98–0.98)	1.06 (1.06–1.06)	1.06 (1.06–1.07)	1.18 (1.18–1.18)	1.08 (1.08–1.08)	1.21 (1.20–1.21)
Generation Y (1977–1995)	0.51 (0.50–0.51)	0.54 (0.54–0.54)	0.63 (0.63–0.63)	0.67 (0.67–0.679)	0.64 (0.64–0.64)	0.68 (0.68–0.68)
Generation Z (1996-)	0.13 (0.13–0.13)	0.12 (0.12–0.12)	0.15 (0.15–0.16)	0.15 (0.14–0.15)	0.16 (0.16–0.16)	0.15 (0.15–0.15)
**Baseline year**						
1995	1 (ref. cat.)	1 (ref. cat.)	1 (ref. cat.)	1 (ref. cat.)	1 (ref. cat.)	1 (ref. cat.)
2000	0.68 (0.68–0.68)	0.65 (0.64–0.65)	0.70 (0.70–0.70)	0.65 (0.65–0.66)	0.69 (0.68–0.69)	0.63 (0.63–0.64)
2005	1.11 (1.10–1.11)	1.10 (1.10–1.11)	1.10 (1.10–1.11)	1.11 (1.10–1.11)	1.09 (1.08–1.09)	1.05 (1.04–1.05)
2010	1.13 (1.13–1.13)	1.34 (1.33–1.34)	1.23 (1.22–1.23)	1.30 (1.30–1.31)	1.21 (1.20–1.21)	1.23 (1.22–1.23)
2015	1.34 (1.33–1.35)	1.51 (1.50–1.52)	1.25 (1.24–1.26)	1.43 (1.42–1.44)	1.23 (1.22–1.23)	1.35 (1.34–1.35)

^1^ IRR: incidence rate ratio, CI: confidence interval. ^2^ Adjusted for generation and baseline year; duration of follow-up (years) constrained to 1. ^3^ Adjusted for generation, baseline year, education and income; duration of follow-up (years) constrained to 1. ^4^ Adjusted for generation, baseline year, education, income, number of children <18 years living at home, country of birth and type of residential area; duration of follow-up (years) constrained to 1.

**Table 4:** Associations of sex with years in paid work, overall and by generation in Sweden from 1995–2015.

		IRR (95% CI) ^1^		
		Unadjusted ^2^	Minimum-Adjusted ^3^	Multivariable-Adjusted ^4^
**Overall**	Men	1 (ref. cat.)	1 (ref. cat.)	1 (ref. cat.)
	Women	0.90 (0.90–0.90)	0.91 (0.91–0.91)	0.89 (0.89–0.90)
**Generation (birth year)**				
Traditionalists (pre-1946)	Men	1 (ref. cat.)	1 (ref. cat.)	1 (ref. cat.)
	Women	0.93 (0.92–0.93)	1.11 (1.11–1.12)	1.15 (1.14–1.15)
Baby Boomers (1946–1964)	Men	1 (ref. cat.)	1 (ref. cat.)	1 (ref. cat.)
	Women	0.94 (0.94–0.94)	0.96 (0.96–0.96)	0.94 (0.94–0.94)
Generation X (1965–1976)	Men	1 (ref. cat.)	1 (ref. cat.)	1 (ref. cat.)
	Women	0.86 (0.86–0.86)	0.88 (0.88–0.88)	0.88 (0.88–0.88)
Generation Y (1977–1995)	Men	1 (ref. cat.)	1 (ref. cat.)	1 (ref. cat.)
	Women	0.87 (0.87–0.87)	0.87 (0.87–0.87)	0.87 (0.87–0.87)
Generation Z (1996-)	Men	1 (ref. cat.)	1 (ref. cat.)	1 (ref. cat.)
	Women	1.04 (1.04–1.05)	1.04 (1.03–1.05)	1.04 (1.03–1.05)

^1^ IRR: incidence rate ratio, CI: confidence interval. ^2^ Duration of follow-up (years) constrained to 1. ^3^ Adjusted for education and income; duration of follow-up (years) constrained to 1. ^4^ Adjusted for education, income, number of children <18 years living at home, country of birth, and type of residential area; duration of follow-up (years) constrained to 1.

## Data Availability

The data used in this study is administered by the Division of Insurance Medicine, Karolinska Institutet, and cannot be made publically. According to the General Data Protection Regulation, the Swedish law SFS 2018:218, the Swedish Data Protection Act, the Swedish Ethical Review Act, and the Public Access to Information and Secrecy Act, these type of sensitive data can only be made available, after legal review, for researchers who meet the criteria for access to this type of sensitive and confidential data. Readers may contact Professor Kristina Alexanderson (kristina.alexanderson@ki.se) regarding the data.

## Supplementary Materials

The following are available online at https://www.mdpi.com/article/10.3390/ijerph18094642/s1.
