# Supplementary material for: Work Participation among Women and Men in Sweden: A Register Study of 8.5 Million Individuals
Source: Int J Environ Res Public Health. 2021 Apr 27;18(9):4642. doi: 10.3390/ijerph18094642 (PMC8123810; doi:10.3390/ijerph18094642)
Supplement: Supplementary file 1 [file ijerph-18-04642-s001.zip › ijerph-1200183-supplementary.pdf]

Online Supplement: Work Participation among Women and Men in Sweden: A Register Study of 8.5 Million Individuals

Katriina Heikkilä[1], Ellenor Mittendorfer-Rutz [1], Kristina Alexanderson[1], Marianna Virtanen[2]

[1] Division of Insurance Medicine, Department of Clinical Neuroscience, Karolinska Institutet, Stockholm, Sweden

[2] School of Educational Sciences and Psychology, University of Eastern Finland, Joensuu, Finland

Supplementary Table S1. Associations of generation and baseline year with years on disability pension, among women and men aged 19-69 in Sweden in 1995-2015

	**IRR (95% CI)[1]** **Mutually adjusted[2]**		**Minimum-adjusted[3]**		**Multivariable-adjusted[4]**	
	**Women**	**Men**	**Women**	**Men**	**Women**	**Men**
**Generation (birth year)**						
Traditionalists (pre-1946)	0.82 (0.82 to 0.82)	0.75 (0.75 to 0.76)	0.78 (0.77 to 0.78)	0.77 (0.76 to 0.77)	0.73 (0.73 to 0.74)	0.74 (0.74 to 0.74)
Baby Boomers (1946-1964)	1 (ref. cat.)	1 (ref. cat.)	1 (ref. cat.)	1 (ref. cat.)	1 (ref. cat.)	1 (ref. cat.)
Generation X (1965-1976)	0.79 (0.79 to 0.80)	0.92 (0.91 to 0.93)	0.75 (0.75 to 0.76)	0.86 (0.85 to 0.86)	0.77 (0.76 to 0.77)	0.86 (0.85 to 0.86)
Generation Y (1977-1995)	0.50 (0.50 to 0.50)	0.50 (0.49 to 0.50)	0.44 (0.44 to 0.44)	0.46 (0.45 to 0.46)	0.45 (0.45 to 0.45)	0.48 (0.48 to 0.49)
Generation Z (1996-)	0.12 (0.11 to 0.12)	0.12 (0.11 to 0.13)	0.10 (0.10 to 0.11)	0.11 (0.11 to 0.12)	0.12 (0.11 to 0.12)	0.13 (0.12 to 0.13)
**Baseline year**						
1995	1 (ref. cat.)	1 (ref. cat.)	1 (ref. cat.)	1 (ref. cat.)	1 (ref. cat.)	1 (ref. cat.)
2000	0.94 (0.92 to 0.95)	0.89 (0.87 to 0.90)	0.93 (0.92 to 0.94)	0.89 (0.88 to 0.91)	0.84 (0.83 to 0.85)	0.83 (0.82 to 0.84)
2005	1.30 (1.26 to 1.33)	1.30 (1.26 to 1.33)	1.32 (1.27 to 1.35)	1.28 (1.24 to 1.31)	1.14 (1.11 to 1.17)	1.14 (1.11 to 1.18)
2010	1.67 (1.60 to 1.74)	1.51 (1.45 to 1.579	1.66 (1.59 to 1.73)	1.47 (1.41 to 1.53)	1.41 (1.36 to 1.48)	1.31 (1.26 to 1.36)
2015	4.29 (3.96 to 4.65)	3.87 (3.58 to 4.18)	4.20 (3.88 to 4.55)	3.79 (3.52 to 4.09)	3.52 (3.26 to 3.81)	3.30 (3.06 to 3.56)

[1] IRR: incidence rate ratio, CI: confidence interval

[2] Adjusted for generation and baseline year; duration of follow-up (years) constrained to 1.

[3] Adjusted for generation, baseline year, education and income; duration of follow-up (years) constrained to 1.

[4] Adjusted for generation, baseline year, education, income, number of children <18 years living at home, country of birth and type of residential area; duration of follow-up (years) constrained to 1.

Supplementary Table S2. Associations of sex with years on disability pension, overall and by generation in Sweden in 1995-2015

		IRR (95% CI)[1] Unadjusted[2]	Minimum-adjusted[3]	Multivariable-adjusted[4]
**Overall**	Men	1 (ref. cat.)	1 (ref. cat.)	1 (ref. cat.)
	Women	0.97 (0.97 to 0.98)	0.97 (0.97 to 0.97)	0.98 (0.97 to 0.98)
**Generation (birth year)**				
Traditionalists (pre-1946)	Men	1 (ref. cat.)	1 (ref. cat.)	1 (ref. cat.)
	Women	1.02 (1.01 to 1.02)	0.97 (0.96 to 0.97)	0.97 (0.97 to 0.98)
Baby Boomers (1946-1964)	Men	1 (ref. cat.)	1 (ref. cat.)	1 (ref. cat.)
	Women	0.93 (0.93 to 0.94)	0.95 (0.95 to 0.96)	0.99 (0.98 to 0.99)
Generation X (1965-1976)	Men	1 (ref. cat.)	1 (ref. cat.)	1 (ref. cat.)
	Women	0.81 (0.80 to 0.81)	0.83 (0.82 to 0.84)	0.89 (0.88 to 0.90)
Generation Y (1977-1995)	Men	1 (ref. cat.)	1 (ref. cat.)	1 (ref. cat.)
	Women	0.94 (0.93 to 0.95)	0.95 (0.94 to 0.97)	0.96 (0.95 to 0.97)
Generation Z (1996-)	Men	1 (ref. cat.)	1 (ref. cat.)	1 (ref. cat.)
	Women	0.96 (0.91 to 1.02)	0.96 (0.91 to 1.02)	0.97 (0.91 to 1.02)

[1] IRR: incidence rate ratio, CI: confidence interval

[2] Duration of follow-up (years) constrained to 1.

[3] Adjusted for education and income; duration of follow-up (years) constrained to 1.

[4] Adjusted for education, income, number of children <18 years living at home, country of birth and type of residential area; duration of follow-up (years) constrained to 1.

Supplementary Table S3. Associations of sex with years in paid work, overall and by generation in Sweden in 1995-2015, among women and men with no children aged <=18 years living at home

		IRR (95% CI)[1] Unadjusted[2]	Minimum-adjusted[3]	Multivariable-adjusted[4]
**Overall**	Men	1 (ref. cat.)	1 (ref. cat.)	1 (ref. cat.)
	Women	0.89 (0.88 to 0.90)	0.90 (0.90 to 0.90)	0.91 (0.91 to 0.91)
**Generation (birth year)**				
Traditionalists (pre-1946)	Men	1 (ref. cat.)	1 (ref. cat.)	1 (ref. cat.)
	Women	0.96 (0.96 to 0.97)	1.17 (1.17 to 1.18)	1.17 (1.17 to 1.18)
Baby Boomers (1946-1964)	Men	1 (ref. cat.)	1 (ref. cat.)	1 (ref. cat.)
	Women	0.95 (0.94 to 0.95)	0.98 (0.98 to 0.98)	0.98 (0.98 to 0.98)
Generation X (1965-1976)	Men	1 (ref. cat.)	1 (ref. cat.)	1 (ref. cat.)
	Women	0.94 (0.94 to 0.94)	0.96 (0.96 to 0.96)	0.96 (0.96 to 0.96)
Generation Y (1977-1995)	Men	1 (ref. cat.)	1 (ref. cat.)	1 (ref. cat.)
	Women	0.86 (0.86 to 0.86)	0.87 (0.87 to 0.87)	0.88 (0.88 to 0.88)
Generation Z (1996-)	Men	1 (ref. cat.)	1 (ref. cat.)	1 (ref. cat.)
	Women	1.06 (1.04 to 1.07)	1.05 (1.04 to 1.06)	1.05 (1.04 to 1.06)

[1] IRR: incidence rate ratio, CI: confidence interval

[2] Duration of follow-up (years) constrained to 1.

[3] Adjusted for education and income; duration of follow-up (years) constrained to 1.

[4] Adjusted for education, income, number of children <18 years living at home, country of birth and type of residential area; duration of follow-up (years) constrained to 1.
